# Antifungal Activities of Wood and Non-Wood Kraft Handsheets Treated with *Melia azedarach* Extract Using SEM and HPLC Analyses

**DOI:** 10.3390/polym13122012

**Published:** 2021-06-20

**Authors:** Mohamed Z. M. Salem, Saqer S. Alotaibi, Wael A. A. Abo Elgat, Ayman S. Taha, Yahia G. D. Fares, Ahmed M. El-Shehawi, Rehab Y. Ghareeb

**Affiliations:** 1 Forestry and Wood Technology Department, Faculty of Agriculture (EL-Shatby), Alexandria University, Alexandria 21545, Egypt; 2 Department of Biotechnology, College of Science, Taif University, P.O. Box 11099, Taif 21944, Saudi Arabia; saqer@tu.edu.sa (S.S.A.); elshehawi@hotmail.com (A.M.E.-S.); 3 Restoration Department, High Institute of Tourism, Hotel Management and Restoration, Abukir, Alexandria 21526, Egypt; watsat20@yahoo.com; 4 Conservation Department, Faculty of Archaeology, Aswan University, Aswan 81528, Egypt; aymansalahtaha82@yahoo.com; 5 Laboratory and Research, Misr Edfu Pulp Writing and Printing Paper Co. (MEPPCO), Aswan 81656, Egypt; yahyagml@yahoo.com; 6 Plant Protection and Biomolecular Diagnosis Department, Arid Lands Cultivation Research Institute (ALCRI), City of Scientific Research and Technological Applications (SARTA, City), New Borg El Arab City, Alexandria 21934, Egypt; reyassin_ghareeb@yahoo.com

**Keywords:** handsheet properties, wood and non-wood raw materials, antifungal activity, pulping, *Melia azedarach* heartwood extract

## Abstract

The main objective of this work was to evaluate pulp produced by kraft cooking for wood materials (WMT) (*Bougainvillea spectabilis*, *Ficus altissima*, and *F. elastica*) and non-wood materials (NWMT) (*Sorghum bicolor* and *Zea mays* stalks) and to study the fungal activity of handsheets treated with *Melia azedarach* heartwood extract (MAHE) solutions. Through the aforementioned analyses, the ideal cooking conditions were determined for each raw material based on the lignin percentage present. After cooking, pulp showed a decrease in the Kappa number produced from WMT, ranging from 16 to 17. This was in contrast with NWMT, which had Kappa numbers ranging from 31 to 35. A difference in the optical properties of the pulp produced from WMT was also observed (18 to 29%) compared with pulp produced from NWMT (32.66 to 35.35%). As for the evaluation of the mechanical properties, the tensile index of the pulp ranged from 30.5 to 40 N·m/g for WMT and from 44.33 to 47.43 N·m/g for NWMT; the tear index ranged from 1.66 to 2.55 mN·m^2^/g for WMT and from 4.75 to 5.87 mN·m^2^/g for NWMT; and the burst index ranged from 2.35 to 2.85 kPa·m^2^/g for WMT and from 3.92 to 4.76 kPa·m^2^/g for NWMT. Finally, the double fold number was 3 compared with that of pulp produced from pulp, which showed good values ranging from 36 to 55. In the SEM examination, sheets produced from treated handsheets with extract from MAHE showed no growth of *Aspergillus fumigatus* over paper discs manufactured from *B. speclabilis* pulp wood. Pulp paper produced from *Z. mays* and *S. bicolor* stalks was treated with 1% MAHE, while pulp paper from *F. elastica* was treated with 0.50% and 1% MAHE. With the addition of 0.5 or 1% MAHE, *Fusarium culmorum* showed no increase in growth over the paper manufactured from *B. speclabilis*, *F. altissima*, *F. elastica* and *Zea mays* pulps with visual inhibition zones found. There was almost no growth of *S. solani* in paper discs manufactured from pulps treated with 1% MAHE. This is probably due to the phytochemical compounds present in the extract. The HPLC analysis of MAHE identified *p*-hydroxybenzoic acid, caffeine, rutin, chlorogenic acid, benzoic acid, quinol, and quercetin as the main compounds, and these were present in concentrations of 3966.88, 1032.67, 834.13, 767.81, 660.64, 594.86, and 460.36 mg/Kg extract, respectively. Additionally, due to the importance of making paper from agricultural waste (stalks of *S. bicolor* and *Z. mays*), the development of sorghum and corn with high biomass is suggested.

## 1. Introduction

There are shortages of conventional raw materials that are used for the production of pulp and paper products. Together with the increasing demand for paper worldwide, this has led to a focus on the use of non-wood fibers or forestry residues as sources of raw materials. There are various lignocellulosic materials that can be produced from agricultural residues from harvesting and pruning operations that can be used for this purpose [1]. Specifically, the most important agricultural residues, due to their abundance, are cereal straw, sunflower stalks, bagasse, waste from the palm oil industry, vine shoots, cotton stems, olives, waste from orange and peach tree pruning, waste from vegetable crops, sorghum, abaca, sisal, jute, hemp, kenaf, flax, linen, bamboo, and papyrus sheets [2,3,4,5,6,7,8,9,10]. Non-wood raw materials are characterized by a low density and more porous structure, with a lower lignin content in most cases. Thus, they require less energy and chemicals to separate fibers during pulp production than wood species do [11]. It was estimated that 8% of commercially produced pulp is non-wood pulp [12].

Several non-wood materials, such as flax plants, dhaincha (*Sesbania bispinosa*), jute stick (*Corchorus capsularis*), sugarcane bagasse, cotton stalk, rice straw, wheatgrass, smooth bromegrass, switchgrass (*Panicum virgatum*), *Miscanthus giganteus*, and bamboo, have been studied to assess their suitability for the production of pulp and paper [7,8,9,10,13,14,15,16,17,18,19,20,21,22]. For example, sorghum stalks (*Sorghum bicolor*) can be used as an alternative source for pulp production. They are characterized by short fibers and a high proportion of fine fibers, so they can be used to produce low luminosity printing and kraft paper [12]. Additionally, it was stated that the production of sorghum stalk pulp has a lower requirement for chemicals than woody pulp production, so it is a suitable method for the production of quality paper [12].

Previous studies have focused on the properties of pulp and paper produced from *Zea mays* and *S. bicolor* stalks and have shown them to have promising values in terms of mechanical properties, such as the tensile index, burst index, tear index, burst index, and double fold number [15,16,22,23,24,25].

*Ficus* trees, belonging to the Moraceae family, could be suitable for use in pulp and paper production. The structural parameters of wood from 12 Nigerian species of *Ficus* have been shown to be suitable for use in pulp and paper production [26] due to, for example, the low vessel count and size of *F. exasperata* wood [27] or the high content of cellulose polymers in *Opuntia ficus-indica* [28].

Most previous studies in this area have shown that the properties of paper produced from softwood species are better than those of paper produced from hardwood species [29]. Studies from Egypt characterized the papermaking properties of woods from trees such as *Populus alba*, *Eucalyptus camaldulensis*, *Meryta sinclairii, Acacia nilotica*, and *Cupressus sempervirens*. *A. nilotica* wood was found to produce a significantly superior net pulp yield [29,30], and *P. alba* wood was shown to produce high-quality paper [29]. Furthermore, the small-diameter logs or branches resulting from the process of pruning fruit or timber trees have been studied in terms of their suitability for the production of pulp and paper [30,31,32,33,34,35].

The kraft and soda–anthraquinone pulping methods are used for hardwood species, for example, for the pulping of *P. tremuloides* clones [36]. For agriculture residues, kraft pulping is used for wheat or barley straw [37,38] and for pruning olive tree wood [31,35]. It is also used for *Leucaena leucocephala*, *Cytisus proliferus*, and *Hesperaloe funifera* [39,40], *Cynara cardunculuns* L. [41], vine shoots [42], and kenaf (*Hibiscus cannabinus* and *H. sabdariffa*) [43].

Natural products, such as phenolics, flavonoids, and essential oils, which can be extracted from medicinal and aromatic plants are used as mediated agents for the green synthesis of nanoparticle materials as well as in adhesives to produce bio-based adhesives or cellulose film, have shown promising antimicrobial activity [44,45,46,47,48,49] and may enhance pulp fibers [50,51].

Different materials have been used to enhance the fungal resistance properties of pulp and paper. For example, *Pinus rigida* ground wood has been used as an additive to linen fiber pulp and has shown resistance against *Aspergillus terreus*, *A. niger*, and *Fusarium culmorum* [7]. Further, nanomaterials have been added to papyrus sheets to guard against *A. flavus*, *A. niger*, and *Colletotrichum gloeosporioides* [8]. Paper discs made from *E. camaldulensis* and *M. sinclairii* branchwood pulp and treated with oily extracts from *Melia azedarach* fruits, *Sinapis alba* seeds, and *Magnolia grandiflora* leaves (in concentrations of 3% and 5%) showed no growth of *A. niger* and *A. terreus* [30].

*Melia azedarach*, or the chinaberry tree, belongs to the mahogany family, Meliaceae. It is a deciduous tree with high-quality, medium-density wood colored from light brown to dark red. The wood has many uses, for example, in construction, furniture, plywood, and boxes [52,53,54]. Heartwood was found to be resistant to white and brown rot fungi [55]. Its extracts showed the presence of bakayanin, bakalactone, and tannin compounds [56], and its stem extracts showed insecticidal activity against *Earias vittella*, *Helicoverpa armigera*, and *Plutella xylostella* [57]. These activities are mostly related to the presence of active chemical compounds, such as phenolic, flavonoid, and limonoid compounds [58]. The technological processing of wood produces a large quantity of waste in the form of saw-dust and shavings that, in the present study, were subsequently used as a source for the extraction of natural chemical compounds.

The present study aimed to evaluate the pulp paper parameters of hardwood species (*Bougainvillea spectabilis, Ficus altissima*, and *F. elastica*), sorghum (*Sorghum bicolor*), and corn (*Zea mays*) stalks using advanced techniques involving the application of *Melia azedarach* heartwood extract to show the enhanced ability of paper discs to act against the growth of three types of mold fungi. This involved the use of SEM.

## 2. Materials and Methods

### 2.1. Preparation of Raw Biomass Materials

**Figure 1:**
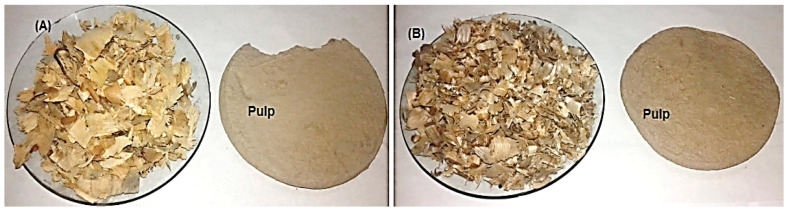

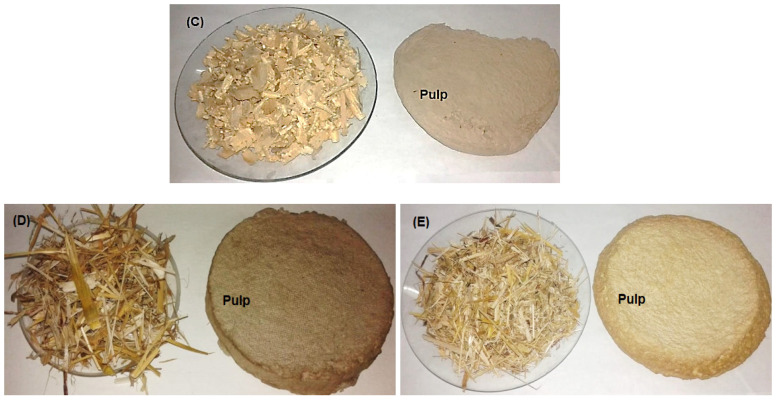
Kraft pulping of wood species and non-wood materials. (**A**) *F. altissima*; (**B**) *F. elastica*; (**C**) *B. spectabilis*; (**D**) *Z. mays*; and (**E**) *S. bicolor*.

Wood from *Bougainvillea spectabilis*, *Ficus altissima*, and *F. elastica*, as well as stalks from *Sorghum bicolor* and *Zea mays*, were collected from Alexandria city in Egypt. Raw materials from *Bougainvillea spectabilis*, *F. altissima*, and *F. elastica* were debarked, chipped, and fractionated using a knife mill, screened, and then cut to small sizes ranging from 2 to 3 cm and homogenized in single lots. Depithed *S. bicolor* and *Z. mays* stalks were cut to chips of sizes ranging from 2.5 to 3 cm (Figure 1). All raw materials were washed with water to remove extraneous dirt, sand, nodes, and other foreign materials. The washed materials were air-dried and then stored in polyethylene bags under dry conditions for use in experimental work.

### 2.2. Chemical Analysis of the Raw Materials

Approximately 20 g raw material samples were ground into powder in a Culatti micro impact mill type grinder (Model MFC, CZ13; ZENITH, Zurich, Germany) with a 1 mm screen, fraction passed through a 40-mesh and retained on +60-mesh to be used for chemical analysis. Chemical characterization of raw materials was conducted as per TAPPI standard test methods. Tests carried out included solvent extraction, measurement of the alcohol–benzene solubility (1:2 *v/v*), and determination of the acid-insoluble lignin, holocellulose, and ash contents using the following methods: TAPPI T204 CM-07 [59], TAPPI T222 OM-15 [60], TAPPI T249-09 [61], and TAPPI T211 OM-16 [62]. All summative chemical analysis results are reported as the percentage of the initial raw biomass material.

### 2.3. Kraft Pulping Procedure Used for Wood Species

Two-hundred-gram oven-dried wood samples from each of *B. spectabilis, F. altissima*, and *F. elastica* were swelled for one day, before being filtrated and impregnated in sodium hydroxide 8% solution for 1 h at 85 °C. Then, they were washed with hot water at 70 °C. Kraft pulping was conducted in a stainless-steel vessel with a 3 L capacity under rotation in an oil bath. The pulping conditions were as follows for all raw wood materials: 17% active alkalinity, 20% sodium oxidate to produce sulfidity, a temperature of 180 °C, a reaction time of 180 min, and a liquor ratio (liquid to wood ratio) of 10:1. The solid residue was defibrated and washed with hot and cold water until reaching a neutral pH. The resultant pulp was screened on a valley flat screen with 0.25 mm slots. All woody species were pulped in triplicate. The yield, residual alkali, kappa number, freeness of pulp (Schopper–Riegler, °SR), ash content in pulp, and the opacity and brightness of pulp were determined by the following TAPPI standard methods: T210 CM-03 [63], T625 CM-14 [64], T236 om-13 [65], ISO 5267-1 [66], T211 OM-16 [62], T425 OM-16 [67], and T452 OM-18 [68].

### 2.4. Kraft Pulping of Corn and Sorghum Stalks

Two-hundred-gram samples of oven-dried raw material from corn and sorghum stalks were swelled for one day, filtrated, and washed several times with hot water. Kraft pulping was conducted in a stainless-steel vessel with a 2 L capacity under rotation in an oil bath for the pulping of corn and sorghum stalks. The following parameters were used: 11% active alkalinity, a temperature of 160 °C, a reaction time of 35 min, and a liquor ratio (liquid to stems ratio) of 10:1. The solid residue was defibrated and washed with hot water and cold water until reaching a neutral pH. The resultant pulp was screened in a valley flat screen with 0.25 mm slots. The pulp collection and conventional bleaching stages were carried out with sodium hydroxide and sodium hypochlorite, and excess distilled water was used for washing between stages (Figure 1). The yield, residual alkali, kappa number, freeness of pulp (Schopper–Riegler, °SR), ash content in pulp, and opacity and brightness of pulp were determined using the following TAPPI standard methods: T210 CM-03 [63], T625 cm-14 [64], T236 OM-13 [65], ISO 5267-1 [66], T211 OM-16 [62], T425 OM-16 [67], and T452 OM-18 [68].

### 2.5. Handsheet Formation and Paper Testing

Handsheet formation was used to prepare standard pulp sheets (200 cm²) for physical testing according to T 220 sp-16 [69]. The samples were diluted to 2000 mL (1.2%) with water and disintegrated at 3000 rpm. After that, the stock was diluted to 0.15% with water at a temperature of 22 °C. To make the sheets, the handsheet formation machine was used to turn on water, and the container was filled with water. Then, 500 mL of stock was added to gain the required consistency and rapidly allow water to move down the cylinder (in 3 ± 1 s) using rotary oscillation for stirring. Immediately after the water had drained from the handsheet, the container was opened, and the sheet was picked with blotting paper, before being dried and weighed to calculate the stock concentration.

After constructing a standard sheet for physical testing (1.22 g), the wet test sheet was placed on a clean mirror-polished plate and covered with another dried blotter ready to receive the next couch blotter and sheet. After that, the sheets were placed in the press template to conduct the first round of pressing at 50 psi for 5 min. Then, the polished plates were removed after first pressing them and placing them on another side, and 50 psi was applied for 2.5 min. Subsequently, the sheets attached to the plate were removed from the press template and placed in drying rings. The sheets were dried according to the standard conditions specified in T 402 sp-08 [70] (23 ± 1 °C and 50 ± 2% RH humidity) until equilibrium moisture was achieved, allowing the sheets to become fully dried in the ring before removing them from the plates. Pulp from standard paper sheet samples (200 cm²) and a grammage of about 60 g/m^2^ was prepared for the determination of dry strength properties according to T 205 sp-02 [71].

The samples were conditioned at 50 ± 2% RH and 23 ± 1 °C according to T 402 sp-08 [70] for at least 4 h. Handsheets were made and their strength properties were tested according to T 220 sp-16 [69]. For the handsheets, the properties tested were the tensile index (T 494 OM-13) [72], tear index (T414 om-12) [73], burst index (T 403 OM-15) [74], and double fold number (T 511 OM-13) [75]. Analysis of the physical strength of pulp was performed according to TAPPI standard methods with standard 60 g/m^2^ sheets.

### 2.6. Antifungal Activity of Handsheets Treated with Melia azedarach Heartwood Extract

*Melia azedarach* heartwood (MAH) was ground to powder using a small laboratory mill. About 150 g of powdered wood was extracted by soaking in 200 mL of methanol solvent for three days [76]. The extract was then filtered through filter paper (Whatman no.1) and concentrated by evaporating the solvent to leave the *M. azedarach* heartwood extract (MAHE). The chemical compounds of the extract were analyzed using Agilent 1260 Infinity HPLC Series (Agilent, Santa Clara, CA, USA), as described in our previous studies [77,78,79,80,81,82,83]. Standard HPLC-grade pyrogallol, quinol, gallic acid, catechol, *p*-hydroxybenzoic acid, caffeine, chlorogenic acid, vanillic acid, caffeic acid, syringic acid, vanillin, *p*-coumaric acid, ferulic acid, benzoic acid, rutin (quercetin-3-*O*-rutinoside), ellagic acid, *o*-coumaric acid, salicylic acid, cinnamic acid, myricetin (3,5,7,3′,4′,5′-hexahydroxyflavone), quercetin, rosmarinic acid, naringenin, and kaempferol were purchased from Sigma-Aldrich (St. Louis, MO, USA).

Before carrying out the antifungal activity method, the handsheets were sterilized, and three concentrations of MAHE (1, 0.5, and 0.25%) were prepared through dilution in dimethyl sulfoxide (Sigma-Aldrich, Darmstadt, Germany) (10% DMSO). The extract solutions were added to handsheets by spraying onto the surface of the paper after most of the water had been removed during sheet formation. Discs (0.9 cm in diameter) from the handsheets treated with the extract solutions were prepared and put over a PDA medium. Then, they were inoculated separately with a 9 mm mycelial disc of the pathogenic fungi from a seven-day-old colony of each of the following three fungal strains: *Fusarium culmorum* Fcu761 (acc# MH355954), *Aspergillus fumigatus* Afu694 (acc# MH355959), and *Stemphylium solani* Ssol382 (acc# MH355956). These were compared with discs treated with 10% DMSO as controls. Each treatment was tested in triplicate. After 14 days of incubation at 26 ± 1 °C, the inhibition zones and growth on discs (in mm) were calculated.

### 2.7. SEM Examination of Paper Sheets Treated with MAHE and Inoculated with Fungi

Examination by scanning electron microscope (SEM) was used to observe the extent of fungal growth on paper discs taken from wood treated and untreated with MAHE and inoculated with each of the three fungi using the JFC-1100E ion sputtering device (model JEOL/MP, JSM-IT200 Series, Tokyo, Japan) at 8 kV [7,8,30].

### 2.8. Statistical Analysis

The chemical properties of the raw materials, the pulp properties, and the mechanical and optical characteristics of *B. spectabilis*, *F. altissima*, *F. elastica*, *Z. mays*, and *S. bicolor* were statistically analyzed using one-way ANOVA [84].

## 3. Results and Discussion

### 3.1. Chemical Analysis of the Raw Materials

Table 1 shows the highly significant differences (*P* < 0.01) between the wood and non-wood raw materials in terms of the chemical parameters of the raw materials (benzene–alcohol extractives, lignin, holocellulose, and ash content).

The results for the chemical compounds of the raw materials (Table 2) show that benzene–alcohol extracts, lignin, holocellulose, and ash content were 1.73–13.16%, 16.66–42.33%, 53.37–63.40%, and 1.66–6.73%, respectively. The holocellulose content in *S. bicolor* stalks was in accordance with the range of values reported in the literature: 71.0% [16], 67.2% [23], 68.0% from depithed extraction [85], 61.6% extracted with 1% NaOH [85], 69.0% from depithed extraction [86], and 61.6% extracted with 1% NaOH [86]. The average pulp yield from *S. bicolor* stalks was 45%, and the average kappa number was 14–18 when 16–20% sodium (NaOH) and a 1.5 h cooking time at 160 °C were used [12]. The holocellulose content in *Z. mays* (62.33%) and *S. bicolor* (63.40%) stalks was higher than that in lotus leaf stalks (53.8%) [87].

The lignin content in wood from *B. spectabilis* (35.87%), *F. altissima* (41.33%), and *F. elastica* (42.33%) was much higher than that in hardwood species (25–35%) [19], *Eucalyptus globulus* (23.9%), *E. nitens* (25.5%), *E. urograndis* (26.6%), red oak (27.7%), cottonwood (21.5%), sweet gum (27.2%), acacia (26.8%), birch (22.8%), red alder (24.4%), maple (25.9%) [33], and the poplar cultivar ‘Hybrid 275’ (18.0%) [20]. Additionally, it was higher than that in softwood species such as European larch (31.2%), birch (26.6%), and pine (26.4%), as well as some other non-wood materials such as tall wheatgrass (13.6%), smooth bromegrass (13.7%), tall fescue (14.0%), switchgrass (17.4%), and *Miscanthus giganteus* (17.8%) [20].

The lignin content in *S. bicolor* (16.66%) and *Z. mays* (18.66%) stalks was in the range of that in non-wood monocotyledon species (9–20%) [19]. The lignin content in *S. bicolor* was equal to that found in depithed and extracted samples (16.1%) [16] and lower than that found in tropical hardwoods (25–35%) [19]. The lignin content in both *S. bicolor* and *Z. mays* was lower than in bamboo (26%) [19], rice hulls (20.44%) [88], and lotus leaf stalks (25.4%) [87], and higher than that in smooth bromegrass (13.7%) and tall fescue (14.0%) [20]. The content was also partially in the range of that reported for switchgrass (17.4%), *Miscanthus giganteus* (17.8%) [20], sugar beet (17.67%) [88], and *S. bicolor* stalks (17.4%) [23]. The ash content was high, especially in *S. bicolor*, but others were within the range of tropical hardwood (1 to 3%) [89].

Among the studied materials, it can be seen from the above results that *Z. mays* and *S. bicolor* stalks had the highest amounts of benzene–alcohol extract and holocellulose. *F. altissima* and *F. elastica* wood showed the highest lignin content, while *S. bicolor* stalks, followed by *B. spectabilis* wood, had the highest ash content.

### 3.2. Chemical Analysis of the Produced Pulp

All pulp properties studied (ash content, residual alkali, Kappa number, screen pulp yield, freeness (Schopper–Riegler, (°SR)) were observed to have highly significant differences (*P* < 0.01) among the studied wood and non-wood raw materials (Table 3). In terms of the pulp properties (Table 4), the ash content, residual alkali, Kappa number, screen pulp yield, freeness (°SR), and rejects were 1.76–13.53%, 1.23–7.75 g/L, 16.66–35.66, 35.33–41.00%, 24.33–33.33 °SR, and 0.13–4.23%, respectively.

The Kappa numbers for *B. spectabilis* (35.66), *F. altissima* (31.33), and *Ficus elastica* (35.33) pulps were higher than those of *E. globulus* (17) [34], poplar cultivar ‘Hybrid 275’ (20.63) [20], *E. camaldulensis* (24), and *M. sinclairii* pulps (18) [30]. Additionally, the Kappa numbers recorded for *Z. mays* and *S. bicolor* pulps were comparable with that recorded for *E. globulus* pulp (17) [34] and lower than that for bamboo (*Gigantochloa scortechinii*) (14.2–18.1) [90]. Kraft pulping of bamboo chips was observed to produce a Kappa number of 17.4 [91], which is nearly comparable with our results for *Z. mays* (16.66) and *S. bicolor* (17.66). Additionally, the value was lower than those for some softwood pulps such as European larch (56.83), birch (23.38), and pine (42.22) [20]. Furthermore, the Kappa numbers for *Z. mays* and *S. bicolor* were slightly higher than those for other non-wood materials such as tall wheatgrass (12.25%), smooth bromegrass (14.42), tall fescue (12.71%), switchgrass (13.71), and *Miscanthus giganteus* (14.31) [20]. Results from the Soda-AQ pulping of rice straw showed Kappa numbers ranging from 12.3 to 26 depending on the mesh size [18].

It can be summarized that pulp produced from *S. bicolor* stalks, *F. altissima* wood, *B. spectabilis/F. elastica* wood, *B. spectabilis* wood, and *Z. mays* stalks showed the highest ash%, residual alkali g/L, screen pulp yield, freeness (°SR), and rejects (%) values, respectively.

### 3.3. Mechanical and Optical Properties of the Produced Handsheets

Table 5 shows that all the mechanical and optical properties (tensile index N·m/g, tear index mN·m^2^/g, burst index kPa·m^2^/g, double fold number, opacity %, and brightness %) among the studied handsheets from wood and non-wood raw materials were statistically highly significant effects (*P* < 0.01).

Table 6 shows that the highest tensile index was observed with handsheets produced from *Z. mays* (47.43 N·m/g), followed by those produced from *S. bicolor* (44.33 N·m/g) and *F. elastica* (40 N·m/g). The highest tear index was found in handsheets produced from *Z. mays* (5.87 mN·m^2^/g), followed those produced from *S. bicolor* (4.75 mN·m^2^/g). Additionally, handsheets produced from pulps of *Z. mays* and *S. bicolor* showed the highest burst index and double fold number with values of 4.76 kPa·m^2^/g and 55 and 3.92 kPa·m^2^/g and 36.33, respectively. The highest opacity percentages were observed for handsheets produced from *F. elastica* wood (99.8%) and *F. altissima* wood (99.1%), while the lowest value was found with handsheets produced from *Z. mays* stalks (93.56%). Handsheets produced from *Z. mays* and *S. bicolor* stalks were observed to have the highest brightness values: 35.33% and 32.66%, respectively. The burst index (3.92 kPa·m^2^/g) of the *S. bicolor* handsheet was higher than that reported for *S. bicolor x* *S. bicolor var. sudanense* pulp grown in Turkey under optimum conditions (3.56 kPa·m^2^/g) [16]. The burst index ranged from 0.16 to 0.29 kPa·m^2^/g in sweet sorghum bagasse [23] and was equal as reported to the values reported in papers that produced pulp from *Populus tremula* via the kraft method (3.85 kPa·m^2^/g) [32]. The unrefined unbleached pulp from *Z. mays* stalks had a tensile index, burst index, tear index, and double fold number of 49.1 N·m/g, 3.80 kN/g, 7.53 mN·m^2^/g, and 86, respectively [22]. In another study, a tensile index of 9.1 N·m/g and a tear index (1.2 N·m/g) were reported for pulp produced from corn stalks [15]. The tensile index of corn stalk pulp ranged from 52 (unbeaten state) to 77.0 N·m/g (beaten state) [24] and from 1.83 to 4.39 N·m/g [23]. For our raw materials, the tear index values were similar to those of other materials such as the oil palm leaf (1.8 N·m^2^/g), date palm rachis (4.4 N·m^2^/g), and palmyra fruit (1.1 N·m^2^/g), while our results for the tensile index were higher than that obtained for the oil palm leaf (7.9 N·m/g) and similar to those obtained for the date palm rachis (1.09 N·m/g) and palmyra fruit (13.8 N·m/g) [15].

The burst index value measured for handsheets from *Z. mays* pulp (4.76 kPa·m^2^/g) was lower than that for corn stalk (6.6 kPa·m^2^/g) but higher than that for date palm rachis (1.32 kPa·m^2^/g) and oil palm leaf (0.9 kPa·m^2^/g) [25]. In the folding test, handsheets from corn stalks produced a value of 55 N, while another study reported a value of 2.5 Nm and compared this with oil palm leaf (1.23 Nm) and oil palm (1.9 Nm) [25].

The paper sheets obtained from olive tree pruning pulp were produced with different degrees of refining and were characterized by their stretch index, burst index, and tear index. All paper sheets reached a stretch index value of between 33 and 39 kNm/kg, a burst index of between 1.5 and 2 kN/g, and a tear index of 0.7–2.5 N·m^2^/g. A high refining degree was not used (<45 °SR) [92,93]. Pulp from empty fruit bunches from oil palm showed a burst index value of 4.17 kN/g and a tear index of 7.20 mN·m^2^/g, for a degree of refining of 47.5 °SR, acceptable for the formation of handsheets [2]. Pulp paper made from sunflower seeds had a burst index of 1.15 kN/g and a tear index of 2.04 mN·m^2^/g [13]. Paulownia wood pulp was used to produce paper sheets with a brightness of 27.4% ISO, a tensile index of 28.87 N·m/g, a burst index of 1.22 kPa.m^2^/g, and a tear index of 1.23 kN·m^2^/g [94]. *H. funifera* pulp showed a tensile index of 83.6 N·m/g, a burst index of 7.34 kN/g, and a tear index of 3.20 mN·m^2^/g [14]. The tensile index of degummed whole cotton stalk pulp ranged from 23.40 to 40.54 N·m/g, and the tear index ranged from 3.95 mN·m^2^/g to 4.52 mN·m^2^/g [17]. The mechanical properties of handsheets produced from *Z. mays* and *S. bicolor* stalk pulps were comparable to those from rice straw: a burst index of 2.43–5.34 kPa·m^2^/g, folding endurance of 35–173, a tear index of 6.49–7.49 mN·m^2^/g, and a tensile index of 38.0–55.2 N·m/g, depending on the mesh size [18]. Another study measured the tensile index (N·m/g), burst index (kPa·m^2^/g), and tear index (mN·m^2^/g) of pulp from the European larch (90.0, 7.8, 8.0), birch (105.8, 7.2, 3.8), pine (103.9, 6.9, 6.1), poplar cultivar ‘Hybrid 275’ (109.0, 8.3, 3.8%), tall wheatgrass (81.6, 6.4, 3.2%), smooth bromegrass (89.0, 7.1, 4.4), tall fescue (85.7, 6.2, 4.4%), switchgrass (68.9, 4.3, 3.7), and *Miscanthus giganteus* (73.4, 4.4, 3.7), respectively [20].

Handsheets from *Z. mays* and *S. bicolor* stalks had the highest values for mechanical properties. Additionally, the folding endurance strength showed that paper from those materials could achieve higher folding numbers than those from the hardwood species investigated in this study. Other studies revealed that *Ficus* species with large amounts of parenchyma have a significantly lower ratio of fibers to non-fibrous tissue [26], leading to a lower yield of pulp and a problem of binding resulting from the ‘fine fibers’ [95].

### 3.4. Antifungal Activity and SEM Examination of MAHE-Treated Handsheets

**Figure 2:**
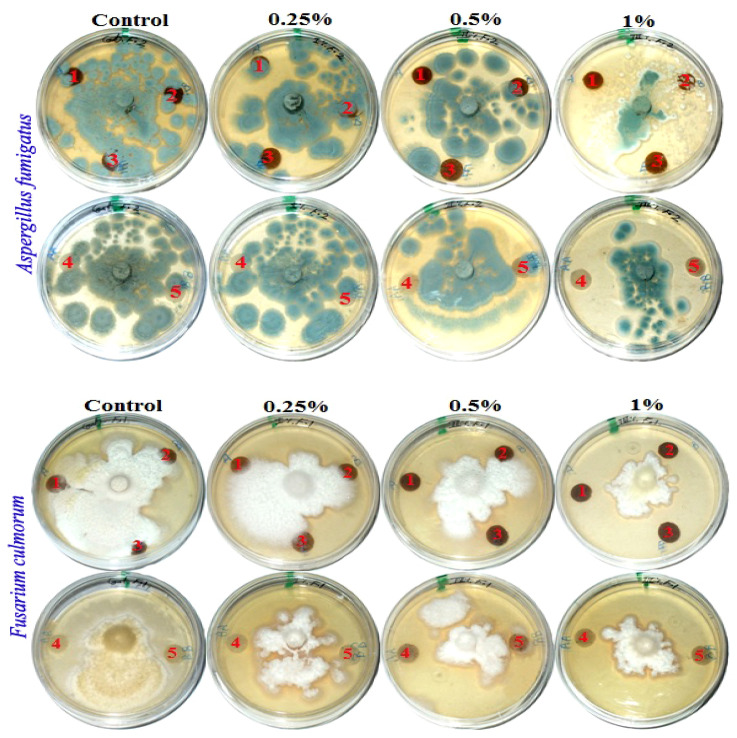

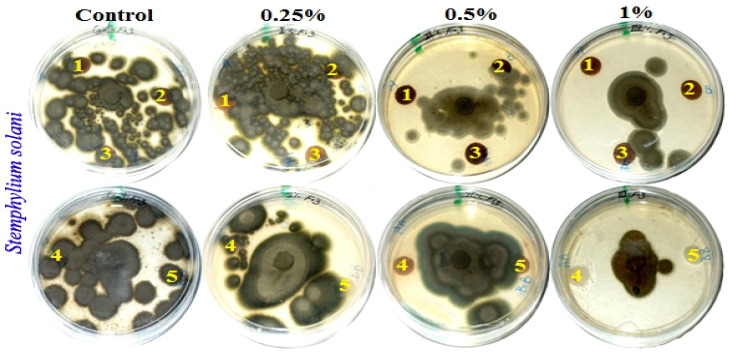
Visual observation of the antifungal activity of paper sheets treated with MAHE; paper sheet samples from pulp produced from (1) *B. speclabilis*; (2) *F. altissima*; (3) *F. elastica*; (4) *Z. mays*; and (5) *S. bicolor*.

Figure 2 presents the antifungal activity of the produced handsheets that were previously treated with MAHE against three mold fungi (*Aspergillus fumigatus*, *Fusarium culmorum* and *Stemphylium solani*). The fungal inhibition zones (in mm) and the fungal growth on the treated discs are presented in Table 7.

Fourteen days after incubation, and with an increase in the extract concentration, the inhibition or suppression of fungal growth occurred. According to visual observations and compared with the control treatments, no fungal growth of *A. fumigatus* occurred on paper discs of *B. speclabilis* pulp wood or pulp paper produced from *Z. mays* and *S. bicolor* stalks and treated with 1% MAHE, or pulp paper produced from *F. elastica* and treated with 0.50% and 1% of MAHE. At MAHE concentrations of 0.50% and 1%, *F. culmorum* showed no growth on paper discs manufactured from *B. speclabilis*, *F. altissima*, *F. elastica,* and *Z. mays* pulp, with visual inhibition zones found (Table 7), while no growth was found following treatment with 1% MAHE on paper discs made from *S. bicolor* pulp. Furthermore, nearly no growth of *S. solani* was found on paper discs manufactured from the raw materials treated with 1% MAHE, and inhibition zones were found.

**Figure 3:**
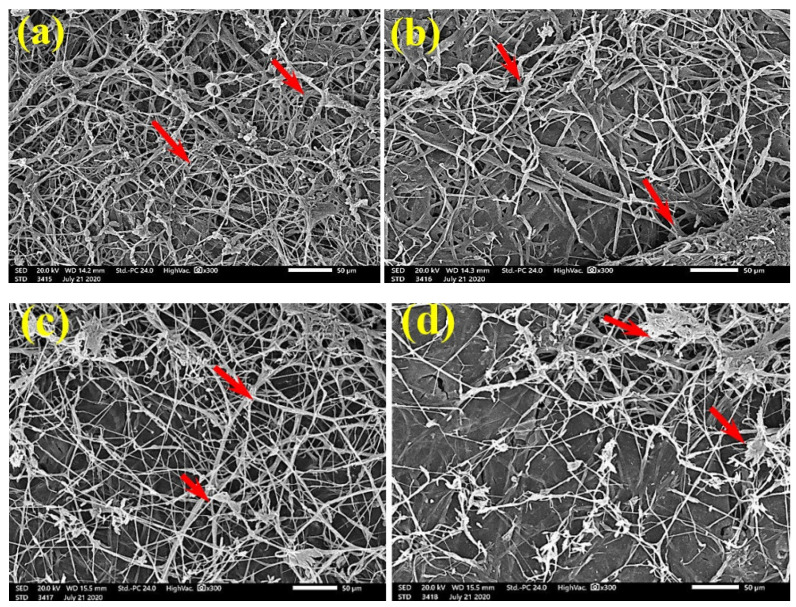

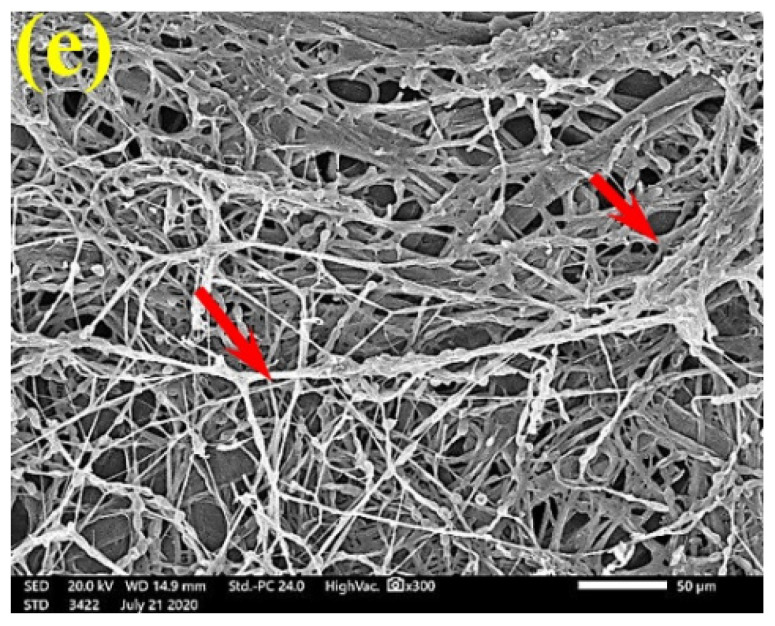
SEM images of the paper sheets manufactured from pulp of (**a**) *Bougainvillea speclabilis*, (**b**) *Ficus altissima*, (**c**) *Ficus elastica*, (**d**) *Zea mays,* and (**e**) *Sorghum bicolor* treated with 10% DMSO (control) and inoculated with *F. culmorum*. Arrows refer to dense growth of fungal mycelia in the sample fibers.

**Figure 4:**
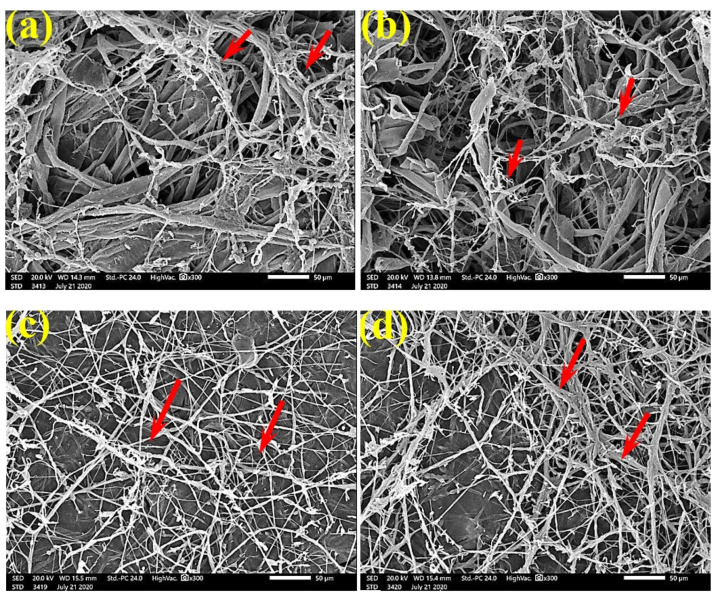
SEM images of the paper sheets manufactured from pulp of (**a**) *Bougainvillea speclabilis*, (**b**) *Ficus altissima*, (**c**) *Ficus elastica,* and (**d**) *Sorghum bicolor* treated with 0.25% *MAHE* and inoculated with *F. culmorum*. Arrows refer to dense growth of fungal mycelia based on the concentration of the extract and the type of paper sheet pulp.

**Figure 5:**
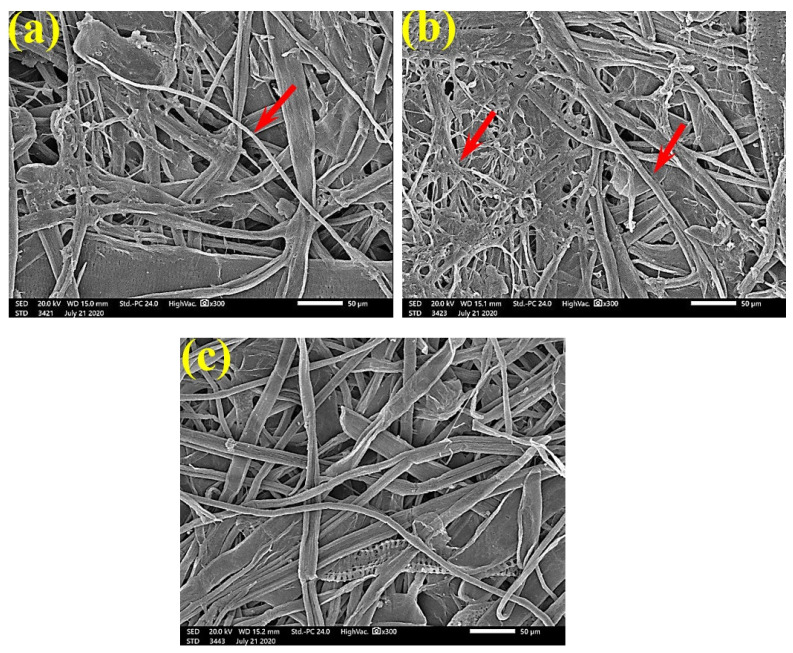
SEM images of the paper sheets manufactured from (**a**) *Ficus altissima*, (**b**) *Sorghum bicolor* treated with 0.5% *MAHE,* and (**c**) *Ficus elastica* treated with 1% *MAHE* and inoculated with *F. culmorum*. Arrows refer to low growth of fungal mycelia based on the concentration of the extract and the type of paper sheet pulp.

**Figure 6:**
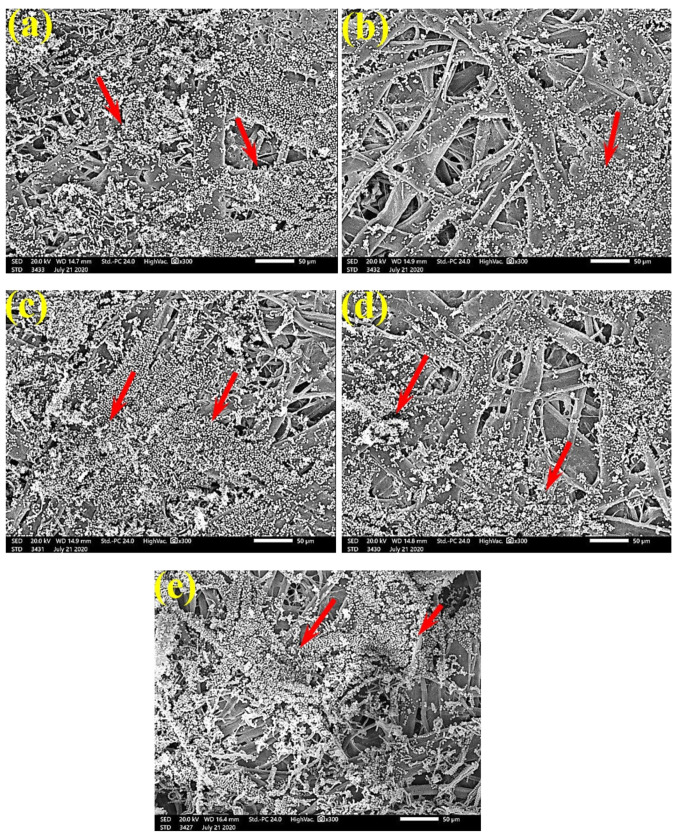
SEM images of paper sheets from (**a**) *B. speclabilis*, (**b**) *F altissima*, (**c**) *F. elastica*, (**d**) *Z. mays,* and (**e**) *S. bicolor* treated with 10% DMSO (control) and inoculated with *A. fumigatus*. Arrows refer to dense growth of fungal mycelia in sample fibers.

**Figure 7:**
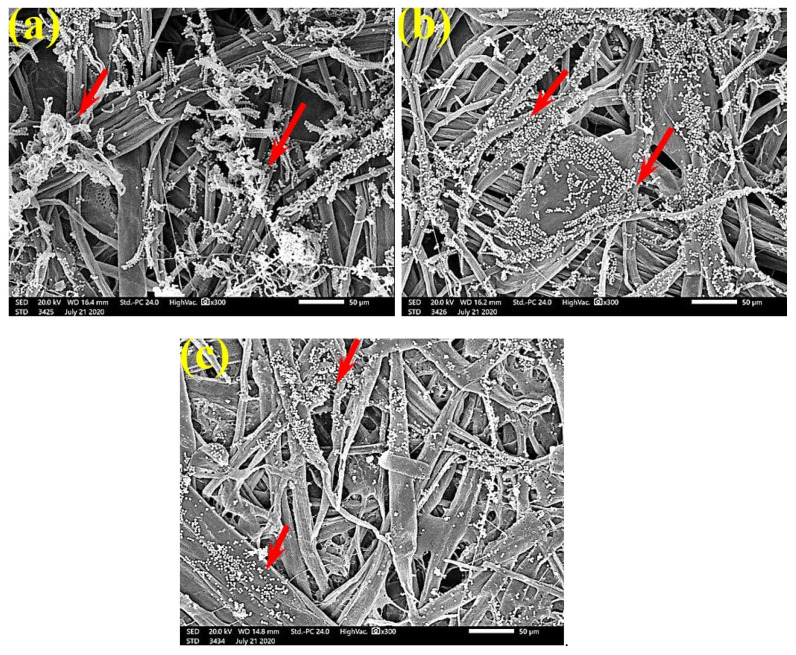
SEM images of paper sheets manufactured from pulps of (**a**) *B. speclabilis*, (**b**) *F. altissima*, and (**c**) *F. elastica* treated with 0.25% MAHE and inoculated with *A. fumigatus*. Arrows refer to dense growth of fungal mycelia based on the concentration of the extract and the type of paper sheet pulp.

**Figure 8:**
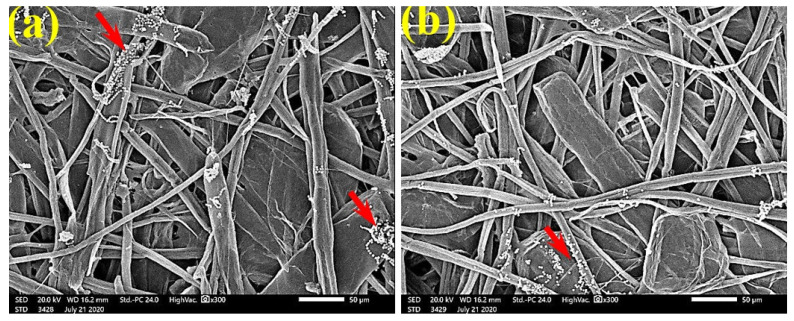

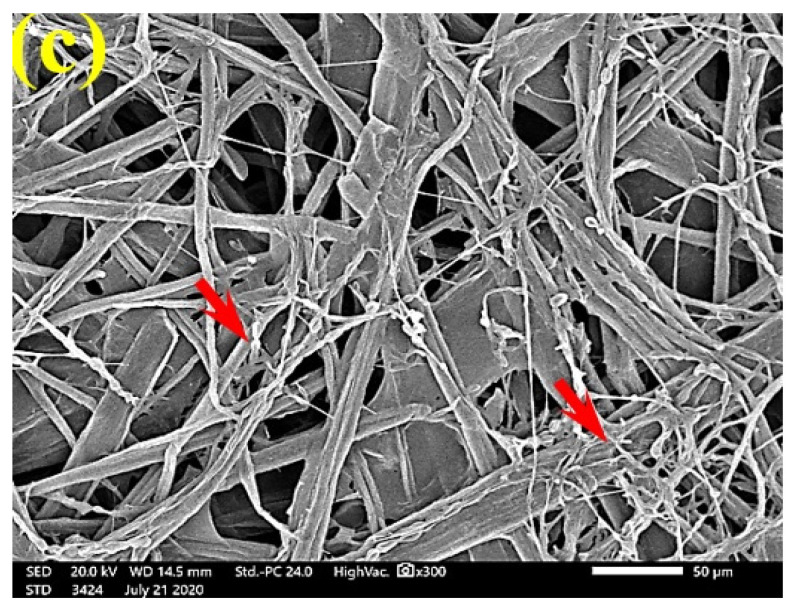
SEM images of paper sheets manufactured from pulps of (**a**) *B. speclabilis*, (**b**) *F. altissima*, and (**c**) *F. elastica* treated with 0.5% MAHE and inoculated with *A. fumigatus*. Arrows refer to low growth of fungal mycelia based on the concentration of the extract and the type of paper sheet pulp.

**Figure 9:**
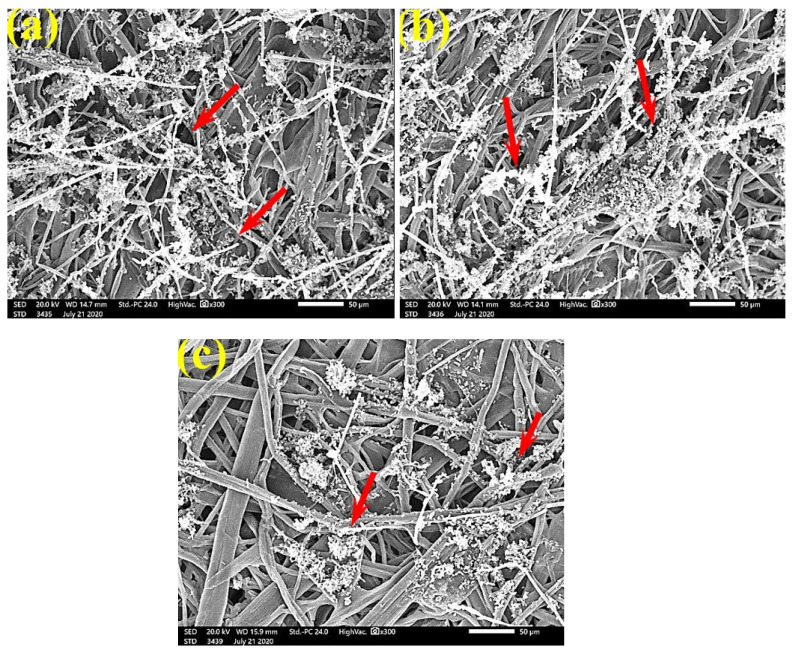
SEM images of the paper sheets manufactured from (**a**) *B. speclabilis*, (**b**) *F. altissima*, and (**c**) *F. elastica* treated with 10% DMSO (control) and inoculated with *S. solani*. Arrows refer to dense growth of fungal mycelia in sample fibers.

**Figure 10:**
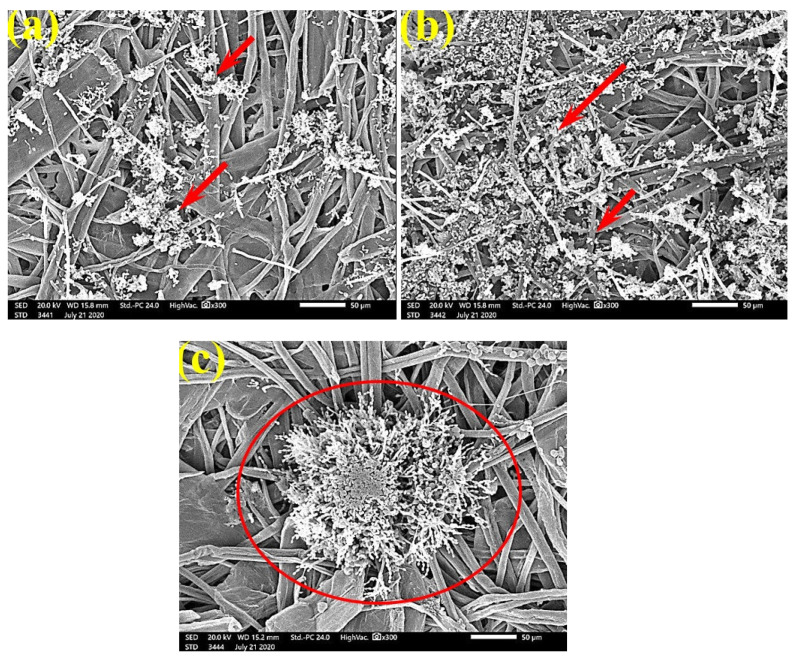
SEM images of paper sheets manufactured from pulps of (**a**) *B. speclabilis*, (**b**) *F. altissima*, and (c) *F. elastica* treated with 0.25% MAHE and inoculated with *S. solani*. Arrows refer to dense growth of fungal mycelia based on the concentration of the extract and the type of paper sheet pulp.

**Figure 11:**
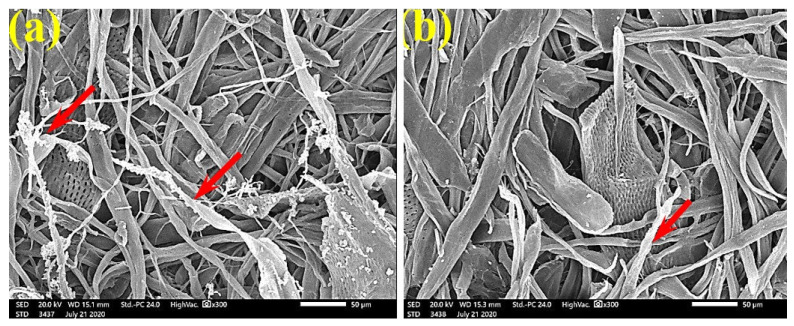
SEM images of paper sheets manufactured from (**a**) *B. speclabilis* and (**b**) *F. altissima* treated with 0.5% MAHE and inoculated with *S. solani*. Arrows refer to low and no growth of fungal mycelia based on the concentration of the extract and the type of paper sheet pulp.

**Figure 12:**
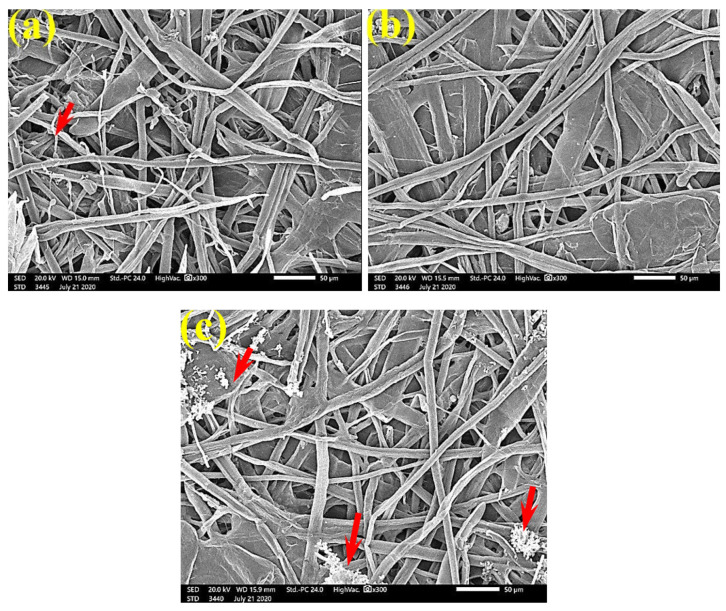
SEM images of the paper sheets manufactured from (**a**) *B. speclabilis*, (**b**) *F. altissima*, and (**c**) *F. elastica* treated with 1% MAHE and inoculated with *S. solani*. Arrows refer to low growth of fungal mycelia based on the concentration of the extract and the type of paper sheet pulp.

SEM examinations of (Figure 3, Figure 4, Figure 5, Figure 6, Figure 7, Figure 8, Figure 9, Figure 10, Figure 11 and Figure 12) the paper discs made from the studied materials, treated with MAHE, and inoculated with the three mold fungi were conducted to observe and show how this MAHE acts against the growth of the studied fungi by suppressing their hyphal and mycelial growth. Figure 3 shows huge, dense fungal mycelia growth (FMG) of *F. culmorum* on the paper discs made from untreated samples (control, 10% DMSO). Additionally, with the application of 0.25% MAHE, dense FMG was still found for *F. culmorum* (Figure 4). With 0.5% MAHE, the FMG of *F. culmorum* was suppressed and the hyphal mass growth was reduced, as shown in Figure 5a,b. Examination of *F. elastica* pulp wood treated with 1% MAHE under SEM found no FMG of *F. culmorum* and fibers were clearly shown (Figure 5c).

Figure 6 shows the dense FMG of *A. fumigatus* on paper discs treated with 10% DMSO (control) made from *B. speclabilis* (Figure 6a), *Ficus altissima* (Figure 6b), *F. elastica* (Figure 6c), *Z. mays* (Figure 6d), and *S. bicolor* pulp (Figure 6e). The FMG of *A. fumigatus* began to decrease following treatment of the paper discs with 0.25% MAHE, as shown in Figure 7. Following treatment with 0.5% MAHE, paper discs made from pulps of *B. speclabilis* (Figure 8a), *F. altissima* (Figure 8b), and *F. elastica* (Figure 8c) showed low FMG of *A. fumigatus*, as the extract suppressed the FMG of *A. fumigatus*, and the fibers were more clearly shown. In the SEM examination, FMG of *S. solani* was clearly shown, with dense growth over the fibers of untreated (control 10% DMSO) handsheets made from *B. speclabilis* (Figure 9a), *F. altissima* (Figure 9b), and *F. elastica* pulp (Figure 9c).

FMG was reduced in the treated handsheets treated with 0.25% *Z. album* extract, but growth was still dense, as *S. solani* mycelia were found over and between the fibers (Figure 10). Low or even no FMG of *S. solani* was found when the handsheets were treated with 0.5% MAHE, and weak interconnections of hyphae with the fiber structure of the paper were found (Figure 11). The FMG of *S. solani* was nearly suppressed (Figure 12a,c) or completely suppressed (Figure 12b) following the application of 1% MAHE to the produced handsheets.

These results are in agreement with those of previous studies [96]. Papyrus strips pretreated with natural extracts were enhanced in terms of the technological (mechanical and optical) and antifungal (against *A. flavus*, *A. niger*, and *C. gloeosporioides*) properties of the produced papyrus sheets [8]. In a previous study, the novel combination of chitosan or Paraloid B-72 c with nanoparticles of Ag, ZnO, or cellulose was used to produce antifungal handsheets. The huge growth of *A. flavus*, *A. terreus*, and *S. solani* that was observed on handsheets produced with pulp with additives of chitosan and Paraloid B-72 at 4% was compared with pulp without additives [9]. The addition of powdered plant materials in the three concentrations was less or not effective at preventing the growth of the three tested fungi—*A. terreus*, *F. culmorum*, and *A. niger*—but treated pulp from *P. rigida* wood had some defense against *A. terreus* and *A. niger* at all tested concentrations [7].

### 3.5. Testing of Phytochemical Compounds of MAHE by HPLC

**Figure 13:**
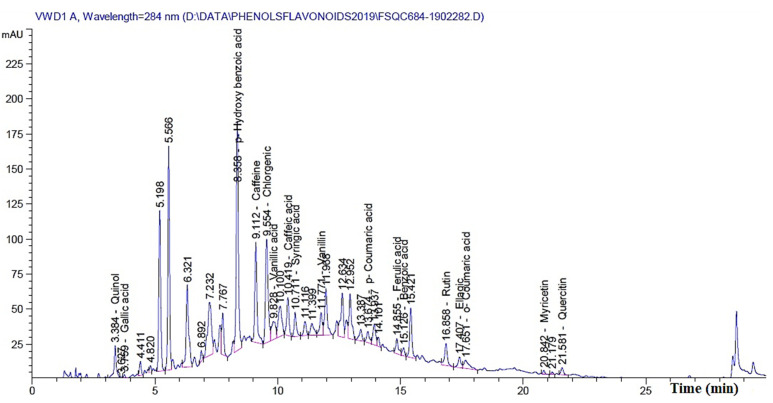
HPLC Fingerprint profile of phytochemicals present in *Melia azedarach* heartwood extract.

The previous results clearly show that by increasing the concentration of MAHE, the suppression of fungal growth occurs. Additionally, some treatments resulted in no fungal growth. These results could be related to the phytochemical compounds present in the extract. Table 8 presents the chemical compounds presented in MAHE. The main compounds (in mg/kg) are *p*-hydroxybenzoic acid (3966.88), caffeine (1032.67), rutin (quercetin 3-*O*-rutinoside) (834.13), chlorogenic acid (767.81), benzoic acid (660.64), quinol (594.86), quercetin (460.36), vanillic acid (366.13), myricetin (302.404), and caffeic acid (130.97). Figure 13 shows the chromatogram peaks that were used to separate the chemical compounds in MAHE.

From different parts of *M. azedarach* (Meliaceae family), phenolic compounds *p*-coumaric acid, vanillic acid, gallic acid, caffeic acid, ferulic acid, protocatechin, *p*-hydroxybenzoic acid, chlorogenic acid, rutin, and salicylic acid were identified. The ingredient components active against *Meloidogyne incognita* were identified as *p*-coumaric acid and *p*-hydroxybenzoic acid [97]. At a wavelength of 280 nm, the main phenolic compounds in the leaves of *Melia azedarach* were found to be rutin, quercetin-3-*O*-neohesperidoside, kaempferol-3-*O*-rutinoside, feruloylglucaric acid, and feruloylquinic acid derivative [98]. Extracts from different parts of *M. azedarach* exhibited fungistatic activity against *A. flavus*, *Diaporthe phaseolorum* var. *meridionales*, *F. oxysporum*, *F. solani*, *F. verticillioides*, and *Sclerotinia sclerotiorum*, where vanillin, 4-hydroxy-3-methoxycinnamaldehyde and (±)-pinoresinol were found to be the main compounds [99]. Additionally, rutin, kaempferol 3-*O*-robinobioside, and kaempferol 3-*O*-rutinoside were isolated from water extract of *M. azedarach* leaves [100]. Gallic acid and (–) epicatechin were isolated from bark extract [101]. Flavonoids naringenin, quercetin, myricetin, and dihydromyricetin were isolated from *Soymida febrifuga* wood extract (Meliaceae family) [102].

## 4. Conclusions

Cellulosic fibers from wood and non-wood raw materials are a renewable material used for production in the pulp and paper industries. In this study, fibers from woody materials (*Bougainvillea spectabilis*, *Ficus altissima*, and *F. elastica*) and stalks of *Sorghum bicolor* and *Zea mays* were used for the production of pulp paper. They were treated with MAHE at concentrations of 0.25%, 0.50%, and 1% and then inoculated separately with the mold fungi *Fusarium culmorum, Aspergillus fumigatus*, and *Stemphylium solani*. A chemical analysis of raw materials was conducted (wood of *Bougainvillea spectabilis*, *Ficus altissima*, *F. elastica* and stalks of *Sorghum bicolor* and *Zea mays*), and the concentration of benzene–alcohol extracts ranged from 1.7 to 4.87% in wood fibers and 5.53 to 13.1% in non-wood fibers. The lignin content ranged from 35.37 to 42.33% in wood fibers and 16.66 to 18.88% in non-wood fibers. The percentage of holocellulose was 35.87 to 42.33% in wood fibers and 62.3 to 63.4% in non-wood fibers. Finally, the ash percentage ranged from 1.66 to 3.43% in wood fibers and 2.73 to 6.73 % in non-wood materials. The mechanical properties of pulp handsheets made from *Z. mays* and *S. bicolor* stalks were much more favorable than those made from hardwoods under the conditions of this study. Additionally, the folding endurance strength showed that paper made from those materials can have a greater folding number than that for paper made from hardwood species. Treatment with MAHE was associated with an improvement in antifungal properties, with visual inhibition zones found and confirmed with SEM examination. The results of the SEM examination of MAHE-treated handsheets inoculated with the tested fungi showed that by increasing the MAHE concentration, the suppression of fungal growth increased. Additionally, with a MAHE concentration of 1%, the three tested fungi showed low or no growth of fungal mycelia depending on the MAHE concentration and the type of handsheet pulp. This indicates the potential for the use of *M. azedarach* heartwood extract to produce pulp paper in an ecofriendly way.

## Figures and Tables

**Table 1:** ANOVA test for the chemical properties of raw materials.

SOV	df	Sum of Squares	Mean Square	F Value
Benzene and Alcohol Extractives				
Species	4	251.8815	62.9703	15,535.5 **
Error	10	0.0405	0.0040	
Lignin				
Species	4	1849.473	462.36	1733.49 **
Error	10	2.667	0.266	
Holocellulose				
Species	4	273.88	68.47	925.20 **
Error	10	0.74	0.074	
Ash content in raw material				
Species	4	47.77	11.94	15,855.1 **
Error	10	0.007	0.0007	

SOV; source of variance, df; degrees of freedom. **: Highly significant at level of probability of 0.05.

**Table 2:** Chemical properties of raw materials.

Biomass	Chemical Properties			
	Benzene–Alcohol Extractives	Lignin (%)	Holocellulose (%)	Ash (%)
*Bougainvillea spectabilis*	4.87 *^c^* ± 0.015 *	35.87 *^c^* ± 0.01	54.56 *^c^*± 0.15	3.43 *^b^* ± 0.015
*Ficus altissima*	2.24 *^d^* ± 0.005	41.33 *^b^* ±0.57	54.73 *^c^* ± 0.05	2.23 *^d^* ± 0.005
*Ficus elastica*	1.73 *^e^* ± 0.057	42.33 *^a^* ±0.57	53.37 *^d^* ± 0.005	1.66 *^e^* ± 0.011
*Zea mays*	13.16 *^a^* ± 0.057	18.66 *^d^* ± 0.57	62.33 *^b^* ± 0.57	2.73 *^c^* ± 0.057
*Sorghum bicolor*	5.53*^b^*± 0.115	16.66 *^e^* ±0.57	63.40 *^a^* ± 0.10	6.73 *^a^* ± 0.005
LSD 0.05	0.1158	0.93	0.49	0.04

* Values are presented as the mean ± SD, and means with same superscript letter (a–d) within the same column are not significantly different according to LSD_0.05_.

**Table 3:** ANOVA test for the chemical properties of the produced pulp.

SOV	df	Sum of Squares	Mean Square	F Value
Ash in pulp				
Species	4	296.462	74.115	8551.81 **
Error	10	0.086	0.008	
Residual alkali (g/L)				
Species	4	108.518	27.129	19,471.0 **
Error	10	0.0139	0.0013	
Kappa number				
Species	4	1070	267.5	802.50 **
Error	10	3.33	0.333	
Screen pulp yield				
Species	4	59.3307	14.8326	24.72 **
Error	10	6.0004	0.60004	
Freeness				
Species	4	166.26	41.56	77.94 **
Error	10	5.333	0.533	
Rejects				
Species	4	40.7106	10.177	5071.93 **
Error	10	0.02006	0.002	

SOV; source of variance, df; degrees of freedom. **: Highly significant at level of probability of 0.05.

**Table 4:** Chemical properties of the pulps yielded.

Pulp Source	Ash (%)	Residual Alkali g/L	Kappa Number	Screen Pulp Yield (%)	Freeness (°SR)	Rejects (%)
*Bougainvillea spectabilis*	2.73 *^c^* ± 0.115 *	6.23 *^c^* ± 0.057	35.66 *^a^* ± 0.57	41.00 *^a^* ± 1.00	33.33 *^a^* ± 0.57	0.36 *^c^* ± 0.05
*Ficus altissima*	2.13 *^d^* ± 0.057	7.75 *^a^* ± 0.01	31.33 *^b^* ± 0.57	37.66 *^b^* ± 0.57	24.33 *^d^* ± 0.57	0.30 *^c^* ± 0.00
*Ficus elastica*	1.76 *^e^* ± 0.057	6.46 *^b^* ± 0.05	35.33 *^a^* ± 0.57	35.33 *^c^* ± 0.57	28.66 *^b^* ± 0.57	0.13 *^d^* ± 0.057
*Zea mays*	6.70 *^b^* ± 0.10	1.23 *^e^* ± 0.01	16.66 *^c^* ± 0.57	35.82 *^c^* ± 0.01	27.33 *^c^* ± 0.57	4.23 *^a^* ± 0.005
*Sorghum bicolor*	13.53 *^a^* ± 0.115	1.64 *^d^* ± 0.01	17.66 *^c^* ± 0.57	37.33 *^b^* ± 1.15	24.33 *^d^* ± 1.15	2.73 *^b^* ± 0.057
LSD 0.05	0.169	0.067	1.05	1.409	1.32	0.081

* Values are presented as the mean ± SD, and means with same superscript letter (a–d) within the same column are not significantly different according to LSD0.05.

**Table 5:** ANOVA analysis of mechanical and optical properties of the produced handsheets.

SOV	df	Sum of Squares	Mean Square	F Value
Tensile index (N·m/g)				
Species	4	665.124	166.281	352.79 **
Error	10	4.7133	0.4713	
Tear index (mN·m^2^/g)				
Species	4	45.93102	11.482	132493 **
Error	10	0.00086667	0.00008	
Burst index (kPa·m^2^/g)				
Species	4	12.741	3.1853	1159.72 **
Error	10	0.02746	0.0027	
Double fold number				
Species	4	7076.266	1769.066	2041.23 **
Error	10	8.666	0.8666	
Opacity %				
Species	4	85.4226	21.355	2464.12 **
Error	10	0.08666	0.00866	
Brightness %				
Species	4	632.2666	158.066	215.55 **
Error	10	7.333	0.7333	

SOV; source of variance, df; degrees of freedom. **: Highly significant at level of probability of 0.05.

**Table 6:** Mechanical and optical properties of the produced handsheets.

Pulp source	Mechanical properties				Optical properties	
	Tensile Index (N·m/g)	Tear Index (mN·m^2^/g)	Burst Index (kPa·m^2^/g)	Double Fold Number	Opacity (%)	Brightness(%)
*B. spectabilis*	30.50 ***^e^***± 0.10 *	1.66 ***^d^***± 0.01	2.52 ***^d^***± 0.01	3***^c^***± 1.00	98.5***^c^***± 0.10	29***^c^***± 1.00
*F. altissima*	32 ***^d^***± 1.00	2.55***^c^***± 0.01	2.35 ***^e^***± 0.01	3 ***^c^***± 1.00	99.1 ***^b^***± 0.10	22 ***^d^***± 1.00
*F. elastica*	40 ***^c^***± 1.00	1.47 ***^e^***± 0.01	2.85***^c^***± 0.01	3 ***^c^***± 1.00	99.8 ***^a^***± 0.10	18 ***^e^***± 1.00
*Z. mays*	47.43 ***^a^***± 0.11	5.87 ***^a^***± 0.01	4.76 ***^a^***± 0.11	55 ***^a^***± 1.00	93.56 ***^e^***± 0.05	35.33 ***^a^***± 0.57
*S. bicolor*	44.33 ***^b^***± 0.57	4.75 ***^b^***± 0.005	3.92 ***^b^***± 0.01	36.33 ***^b^***± 0.57	95.4 ***^d^***± 0.10	32.66 ***^b^***± 0.57
LSD 0.05	1.249	0.0169	0.0953	1.693	0.1694	1.557

*: Means with the same superscript letter (a–d) within the same column are not significantly different according to LSD_0.05_.

**Table 7:** Visual screenings of the antifungal activity of paper sheets treated with MAHE against the growth of *Aspergillus fumigatus*, *Fusarium culmorum,* and *Stemphylium solani* 14 days after inoculation.

Pulp Paper.	MAHE Con.	*Aspergillus fumigatus*		*Fusarium culmorum*		*Stemphylium solani*	
		Inhibition Zone (mm)	Growth on Disc (mm)	Inhibition Zone (mm)	Growth on Disc (mm)	Inhibition Zone (mm)	Growth on Disc (mm)
*Bougainvillea speclabilis*	10% DMSO	0	4–5	0	4–8	0	5–7
	0.25%	0	8–9	0	1–3	0	4–5
	0.50%	0–1	0–1	6–7	0	2–3	0
	1%	5–6	0	5–9	0	9–10	0
*Ficus altissima*	10% DMSO	0	4–7	0	5–8	0	7–8
	0.25%	0	7–8	0	1–3	0	5–8
	0.50%	0	5–6	0–2	0	0	3–4
	1%	0	1–4	2–4	0	9–11	0
*Ficus elastica*	10% DMSO	0	7–9	0	5–9	0	8–9
	0.25%	0–2	0–2	0	1–4	0–1	0–3
	0.50%	1–2	0	4–5	0	3–4	0
	1%	4–8	0	3–5	0	0–1	0–2
*Zea mays*	10% DMSO	0	3–5	0	3–6	0	9
	0.25%	0	3–4	0–2	0–1	0	8–9
	0.50%	0	0–1	6–8	0	1–3	0
	1%	10–11	0	2–7	0	9–11	0
*Sorghum bicolor*	10% DMSO	0	4–5	0	4–6	0	8–9
	0.25%	0	3–5	0–4	0–2	0	7–9
	0.50%	0	0–1	0–2	0–1	0	5–7
	1%	4–5	0	5–6	0	4–8	0

**Table 8:** Chemical compounds in *Melia azedarach* heartwood extract.

RT (min)	Compound	Amount (mg/Kg)
3.100	Pyrogallol	ND
3.384	Quinol	594.86
3.759	Gallic acid	17.07
5.900	Catechol	ND
8.358	*p*-Hydroxybenzoic acid	3966.88
9.112	Caffeine	1032.67
9.554	Chlorogenic acid	767.81
9.828	Vanillic acid	366.13
10.419	Caffeic acid	130.97
10.711	Syringic acid	117.67
11.771	Vanillin	111.09
13.674	*p*-Coumaric acid	38.61
14.855	Ferulic acid	83.69
15.126	Benzoic acid	660.64
16.858	Rutin (quercetin 3-*O*-rutinoside)	834.13
17.407	Ellagic acid	93.26
17.651	*o*-Coumaric acid	57.49
19.200	Salicylic acid	ND
20.300	Cinnamic acid	ND
20.842	Myricetin	302.404
21.581	Quercetin	460.36
21.900	Rosmarinic acid	ND
22.200	Naringenin	ND
24.000	kaempferol	ND

RT; retention time (min), ND; not detected.

## Data Availability

Not applicable.
